# Meaning of life questionnaire (MLQ) in peruvian undergraduate students: study of its psychometric properties from the perspective of classical test theory (CTT)

**DOI:** 10.1186/s40359-022-00913-6

**Published:** 2022-08-24

**Authors:** Aaron Travezaño-Cabrera, Lindsey W. Vilca, Julisa Quiroz-Becerra, Samy L. Huerta, Rosali Delgado-Vallejos, Tomás Caycho-Rodríguez

**Affiliations:** 1 Departamento de Psicología, Universidad Peruana Unión, Lima, Perú; 2 South American Center for Education and Research in Public Health, Universidad Norbert Wiener, Lima, Perú; 3 Center for Advanced Studies in Psychometrics, Lima, Perú; 4 Facultad de Ciencias de la Salud, Universidad Privada del Norte, Lima, Perú

**Keywords:** Meaning of life, Presence of meaning of life, Search for meaning of life, Undergraduate students

## Abstract

**Background:**

The study of the meaning of life is essential since it plays a protective role in the mental health of university students. However, no studies have shown the adequate psychometric functioning of the MLQ in Latin American university students. For this reason, this research aims to evaluate the internal structure using CTT models, obtain evidence of validity based on the relationship with other variables, perform factorial invariance according to gender, and estimate the reliability of the MLQ.

**Methods:**

A sample of 581 Peruvian undergraduate students of both sexes (29.5% men and 70.5% women) between the ages of 18 and 35 (*M* = 22.6; SD = 3.3) was collected. Along with the MLQ, other instruments were applied to measure satisfaction with life (SWLS), subjective well-being (WBI), and depression (PHQ-9).

**Results:**

In the present study was evidenced that the model of two related factors of nine items presents better adjustment indices (RMSEA = .075; SRMR = .059; CFI = .97; TLI = .96) compared to other models. Also, it was shown that the factorial structure of the MLQ is strictly invariant for the group of men and women. It was also shown that the presence of meaning was positively related to satisfaction with life (.63) and well-being (.60) and negatively to depression (− .56). In contrast, the search for meaning was not significantly related to life satisfaction (− .05) and well-being (− .07); but yes, to depression (.19).

**Conclusion:**

It is concluded that the MLQ from the perspective of CTT has shown adequate evidence of reliability and validity. Therefore, it could be used in future studies and evaluation and intervention processes. In addition, the study provides the first evidence of the psychometric functioning of the scale in university students from Latin America.

## Introduction

The meaning of life refers to how people give meaning to their lives, such as the daily pursuit of goals, and implies the ability to strive for happiness [1–3]. In addition, this construct is a cognitive process that allows us to understand life, oneself, and others comprehensively and consistently [4–6]. Steger [7] adds that the meaning of life integrates two fundamental processes: (a) understanding, which is the ability to find meaning in lived experiences, and (b) coherence, which is the ability to commit and achieve long-term goals.

On the other hand, this construct has been studied from two main factors: (a) presence of meaning of life, is experienced when the person understands himself, the world and identifies his purpose in it [4, 6], and (b) search for meaning, understood from two perspectives: first, as the absence or deficit of meaning [8, 9] and second, as a motivational force and fundamental psychological need of the human being to understand his existence [1, 10]. In this regard, Steger et al. [11] suggests that the search will be healthy according to the internal motivations of each person; that is, it will be positive for those who consider it important to achieve their development, while negative for those who appreciate it as an indicator of low personal growth. In this sense, both factors (presence and search) are inversely related, stronger in individuals with less open-mindedness [11].

Several studies show that the meaning of life plays a fundamental role in people since it reduces the tendency to feel easily upset, moody, and anxious [12]. It is also related to a lower presence of indicators of depression [13], anxiety [14], and post-traumatic distress. Regarding the university population, various studies show that it is a factor that favors academic performance since it allows them to experience less demotivation [15]. Likewise, it improves coping with stressful situations [16] and allows prosocial actions [17]. Other studies show that the meaning of life is linked to happiness [4], optimism [18], satisfaction with life [19], gratitude [20], adaptation to stressors [21], prosocial behavior [17, 22], tolerance for uncertainty [12], and hope [13, 23].

Therefore, the study of this construct is critical because it plays a fundamental role in the mental health of university students, and therefore, it is necessary to have instruments that adequately measure this construct. Concerning this, there are various instruments among the most used are: The Life Regard Index [24], Purpose in Life Test [3], Personal Meaning Index [25], and Meaning in Life Index [26]*.* The Meaning of Life Questionnaire (MLQ) by Steger et al. [6] is the most widely used, made up of 10 items that measure the meaning and significance of the person's life and existence. The MLQ comprises two dimensions: the presence of meaning in life (MLQ-P) and the search for meaning in life (MLQ-S).

Table 1 shows that multiple adaptations of the MLQ have been made in countries such as Japan [27], Argentina [28], Turkey [29], South Africa [19], China [30], Brazil [31], India [32], Australia [33], Nigeria [34], Italy [35], Iran [36] y Ghana [37].

**Table 1:** Psychometric studies of the meaning of life questionnaire (MLQ) in various samples

Study	Sample	Country	N	Model	CFA				Reliability (α)	
					GFI	CFI	RMSEA	SRMR	MLQ-P	MLQ-S
[28]	Adults	Argentina	707	Bidimensional	.94	.95	.09	–	.82	.88
[29]	Adults	Turkey	584	Bidimensional	–	.96	.07	.05	.88	.90
[19]	University students	South Africa	326	Bidimensional	–	.95	.08	–	.85	.84
[32]	Adults	India	550	Bidimensional	.94	.94	.08	–	.78	.81
[33]	Adolescences	Australia	135	Bidimensional		.92	–	.10	.82	.84
[65]	Older adults	Australia	341	Bidimensional	–	.96	.11	.04	–	–
[34]	Adults	Nigeria	809	Bidimensional	.91	.94	.07	–	.82	.86
[35]	Adults	Italy	364	Bidimensional	–	.99	.06	.06	.84	.90
[36]	Patients with cancer and sclerosis	Iran	301	Bidimensional	–	.99	.07	.05	.84	.88
[37]	Adults	Ghana	420	Bidimensional	–	.92	.08	.05	–	–
[57]	High School students	China	1089	Bidimensional	.92	.93	.10	–	.84	.88

Concerning the psychometric properties of the MLQ, the original study [6] carried out on university students showed a two-dimensional structure through confirmatory factor analysis (CFA) whose adjustment indices were satisfactory (CFI = 0.93, TLI = 0.91, RMSEA = 0.09) and presented good reliability values in its dimensions: MLQ-P (*α* = 0.82) y MLQ-S (*α* = 0.87). These results have been corroborated in different investigations [19, 28, 29, 32–35] where a CFA supports the two-dimensional structure with adequate fit indices (CFI > 0.90, TLI > 0.90 and RMSEA < 0.08), as well as appropriate reliability values for both dimensions *α* > 0.77.

Regarding Latin America, the first evaluation of the psychometric properties of the MLQ was carried out in Argentina, where it was found that by eliminating item 9, the adjustment in the two-dimensional structure was adequate for the samples of adults (CFI = 0.95, TLI = 0.92, RMSEA = 0.09) and adolescents (CFI = 0.94, TLI = 91, RMSEA = 0.08). Regarding its reliability, it was shown that both dimensions were greater than 0.80 [28]. A second study conducted in Brazil proposed an orthogonal model of the MLQ, where the dimensions of MLQ-P (CFI = 0.99, TLI = 0.99, RMSEA = 0.075) and MLQ-S dimension (CFI = 0.97, TLI = 0.95, RMSEA = 0.149) presented adequate fit indices. Regarding reliability, the values ​​were satisfactory in the MLQ-P (*α* = 0.90) y MLQ-S (*α* = 0.90) [31]. These findings show no agreement on the internal structure of the scale in the Spanish-speaking population. Furthermore, it is essential to point out that only these two studies have analyzed the internal structure of the MLQ in Latin America. In a university population, no evidence of the psychometric functioning of the scale has been found.

Also, it is essential to point out that several studies show that the correlations between the MLQ-P and MLQ-S factors differ according to culture. In Nigeria [34], Iran [36], China [30], and Japan [27], there is a positive association between both dimensions, which in turn, they vary in their magnitude (0.21 to 0.72). While in countries such as Italy [35], Turkey [29], India [32], Argentina [28], the United States [6], South Africa [19] and Australia [33] show a negative relationship in different magnitudes (− 0.12 to − 0.49).

Due to the above, the lack of psychometric studies that confirm the internal structure of the MLQ in Latin America in the undergraduate students' population is evident. For this reason, this research aims to evaluate the internal structure using CTT models, obtain evidence of validity based on the relationship with other variables, perform factorial invariance according to gender, and estimate the reliability of the MLQ in Peruvian university students. In addition, the following hypotheses were proposed: (a) The MLQ scale presents adequate adjustment indices, (b) The scale presents factorial invariance according to the sex of the participants, and (c) The scale is positively related to life satisfaction and well-being; and is negatively related to depression.

## Method

### Participants

The sample consisted of 581 Peruvian university students, of which 231 were men (29.5%) and 350 were women (70.5%) with an age range of 18 to 40 years (*M* = 22.6, *SD* = 3.3). The vast majority (51.9%) lived with their parents, 18.8% lived alone with their mother, 3.1% only with their father, 11.4% lived with other relatives, 3.6% lived with their friends, and 11.2% lived alone. Regarding the type of university, 91.2% belonged to private institutions and 8.8% to public institutions. Only 42.2% reported working, while 57.8% did not work.

### Instruments

#### Meaning in life questionnaire (MLQ)

The Questionnaire was developed in the United States by Steger et al. [6] and adapted to Spanish in Argentina by Góngora et al. [28]. This scale comprises ten items that measure two dimensions: the presence of meaning (1,4,5,6,9) and the search for meaning (2,3,7,8,10). In addition, the items present seven Likert-type response categories: (1) Absolutely false, (2) Mostly false, (3) Somewhat false, (4) Neither true nor false, (5) Somewhat true, (6) Mostly true, and (7) Absolutely true. Concerning the psychometric properties, in the study by Góngora et al. [28], the scale has validity based on the internal structure (CFI = 0.95, TLI = 0.92, RMSEA = 0.09). It also presents adequate levels of internal consistency for the dimensions of the presence of meaning (*α* = 0.78) and the search for meaning (*α* = 0.80).

#### Satisfaction with life scale (SWLS)

The Spanish version of Atienza et al. [38] was used to assess the global judgment that people make about their level of satisfaction with life. The scale is made up of five items with five response categories ranging from (1) totally disagree to (5) totally agree. Regarding the psychometric properties, the one-dimensional model presented adequate fit indices (GFI = 0.98, NFI = 0.99, NNFI = 0.99) and an adequate level of internal consistency (*α* = 0.84).

#### Weil-being index (WBI)

The version adapted to Peru by Caycho-Rodríguez et al. [39] was used in the study. The scale aims to assess the subjective well-being of the person, for which it has five items with 4 Likert-type response categories, ranging from (0) never, (1) sometimes, (2) many times, and (3) forever. In relation to its psychometric properties, the scale showed validity based on the internal structure in the unidimensional model (CFI = 0.99, RMSEA = 0.053, SRMR = 0.018) and in terms of reliability, adequate values were obtained (*α* = 0.85, *ω* = 0.88).

#### Patient health questionnaire (PHQ‑9)

The version adapted by Villarreal-Zegarra et al. [40] was used, which evaluates indicators of depressive symptomatology during the last two weeks. This questionnaire is made up of nine items with 4 Likert-type response categories: (0) nothing, (1) several days, (2) more than half the days, (3) almost every day. Regarding its psychometric properties, the one-dimensional model showed adequate adjustment indices (CFI = 0.94, TLI = 0.91, RMSEA = 0.089, SRMR = 0.039). Regarding reliability, adequate values were obtained in internal consistency (*α* = 0.87, *ω* = 0.87).

### Procedure

In the present study, the standards of the Helsinki declaration [41], and authorization was obtained from the Ethics Committee of the Universidad Peruana Unión (2021-CE-FCS-UPeU-00230). In the initial phase, content validity was performed by five judges, who evaluated the relevance, coherence, and clarity of the items in the Peruvian context. Later, an apparent validity was performed with 18 students, who evaluated the degree of clarity and were asked to leave recommendations to understand the items better obtaining the suggestions of the two groups, some modifications were made to the items. Finally, for the data collection phase, a virtual form was developed using the Google Forms platform due to the restrictions imposed by the Peruvian Government due to COVID-19. In the first part of the form, the informed consent of the participants was obtained, where the study's objective and the anonymity of the responses were explained. Only the participants who marked the option of agreement agreed and continued to fill out the other parts of the form. The application of the form was carried out in the virtual classrooms of the students.

### Data analysis

In Confirmatory Factor Analysis (CFA), because the items presented more than five response categories [42], the Robust Maximum Likelihood estimator was used [43]. To evaluate the fit of the models, the RMSEA, SRMR, CFI, and TLI indices were used. For the RMSEA and SRMR indices, values less than 0.08 were considered acceptable [44]. For the CFI and TLI indices, values greater than 0.95 were considered adequate [45]. To evaluate the reliability of the scale, Cronbach's alpha coefficient [46] and omega coefficient [47] was used, where a value greater than 0.70 is adequate [48].

The Multi-group Confirmatory Factor Analysis (MGCFA) was used to evaluate the factorial invariance of the scale according to sex, where a sequence of hierarchical variance models was proposed. First, configural invariance (reference model) was evaluated, followed by metric invariance (equality of factor loadings), scalar invariance (equality of factor loading and intercept), and finally, strict invariance (equality of factor loadings, intercept, and residuals). To compare the sequence of models, a formal statistical test was first used, for which the chi-square difference (Δ*χ*2) was used, where non-significant values ​​(*p* > 0.05) suggest invariance between groups. Second, a modeling strategy was used, for which the differences in the CFI (ΔCFI) were used, where values ​​less than < 0.010 show the invariance of the model between the groups [49]. The RMSEA (ΔRMSEA) was also used, where differences less than < 0.015 demonstrate the invariance of the model between the groups [49].

All statistical analyzes were performed using the "lavaan" package for the CFA [50], the "semTools" package for factorial invariance [51]. In all cases, the R Studio Team [52] environment was used for r [53].

## Results

### Content based validity

Table 2 shows the judges' evaluation using the criteria of clarity, relevance, coherence, and context, where the values were adequate (V > 0.70). In addition, modifications were made to the items following the judges' suggestions (item 1, item 4, item 5, item 6, item 7, item 9, and item 10). On the other hand, the apparent validity was carried out, where the focus group of university students indicated that the items were understandable and clear.

**Table 2:** Content validity

Items	Argentine version	Adapted version	V(Rele)	V(Cohe)	V(Clar)	V(Cont)
1	Sé cuál es el sentido de mi vida	Sé cuál es el propósito de mi vida	0.94	0.94	0.83	0.94
2	Estoy buscando algo que me haga sentir que vivo una vida significativa	Estoy buscando algo que me haga sentir que vivo una vida significativa	1.00	1.00	0.94	1.00
3	Siempre estoy buscando encontrar el propósito de mi vida	Siempre estoy buscando encontrar el propósito de mi vida	1.00	1.00	0.94	1.00
4	Mi vida tiene un sentido claro de propósito	Mi vida tiene un propósito claro	1.00	1.00	0.83	0.94
5	Tengo bien en claro qué es lo que hace que mi vida tenga sentido	Sé muy bien lo que da sentido a mi vida	0.83	0.83	0.72	0.78
6	Descubrí un propósito de vida que me da plena satisfacción	Descubrí el propósito de mi vida, el cual me da plena satisfacción	1.00	1.00	0.94	1.00
7	Siempre estoy buscando algo que me haga sentir que mi vida tiene sentido	Siempre estoy buscando algo que me haga sentir que mi vida tiene un propósito	0.83	0.83	0.78	0.78
8	Estoy en la búsqueda de un propósito o misión para mi vida	Estoy en la búsqueda de un propósito o misión para mi vida	1.00	1.00	1.00	1.00
9	Mi vida no tiene un claro propósito	Mi vida no tiene un propósito claro	0.83	0.78	0.78	0.78
10	Estoy buscándole sentido a mi vida	Estoy buscando un significado para mi vida	0.83	1.00	0.78	0.83

*Note* Modifications to items are in bold

### Descriptive analysis

Table 3 shows that item 5 ("I know very well what gives meaning to my life") and item 1 ("I know what the purpose of my life is") present the highest average scores in the sample. That is, most of the participants agree with these statements. It can also be seen that item 10 ("I am looking for meaning in my life") has the lowest average score; that is, most of the participants are undecided regarding this statement ("Neither true nor false"). Regarding the asymmetry and kurtosis indices, it can be seen that all the items present adequate indices (As <  ± 2; Ku <  ± 7), according to the Finney et al. [54] criteria.

**Table 3:** Descriptive analysis of the items

Items	*M*	*SD*	*g1*	*g2*
1. Sé cuál es el propósito de mi vida	5.71	1.33	− 1.45	5.42
2. Estoy buscando algo que me haga sentir que vivo una vida significativa	4.77	1.79	− 72	2.59
3. Siempre estoy buscando encontrar el propósito de mi vida	4.79	1.78	− 71	2.53
4. Mi vida tiene un propósito claro	5.64	1.33	− 1.34	5.09
5. Sé muy bien lo que da sentido a mi vida	5.78	1.29	− 1.28	4.83
6. Descubrí el propósito de mi vida, el cual me da plena satisfacción	5.44	1.44	− 1.08	3.94
7. Siempre estoy buscando algo que me haga sentir que mi vida tiene un propósito	4.79	1.79	− 74	2.61
8. Estoy en la búsqueda de un propósito o misión para mi vida	4.93	1.73	− 79	2.80
9. Mi vida no tiene un propósito claro	5.19	1.83	− 74	2.39
10. Estoy buscando un significado para mi vida	4.19	1.92	− 40	1.97

*M*  Mean, *SD *Standard deviation, *g1* Skewness, *g2*  Kurtosis

### Validity based on internal structure

Table 4 shows that the original model of the scale (model 1a) does not show adequate fit indices (RMSEA = 0.081 [IC90% [0.067—0.095]_;_ SRMR = 0.074; CFI = 0.96; TLI = 0.95). Similarly, the two unrelated factor model (model 1b) does not fit the data (RMSEA = 0.083 [IC90% [0.070–0.097]_;_ SRMR = 0.106; CFI = 0.96; TLI = 0.94). In the two previous models, it can be seen that item 9 shows a low factorial weight (*λ* < 0.49) with a high level of associated error (e5 > 0.75). Faced with this, item 9 was removed, and a model of two related factors of nine items was proposed (model 2a), which presented adequate adjustment indices (RMSEA = 0.075 [IC90% 0.059 – 0.092]_;_ SRMR = 0.059; CFI = 0.97; TLI = 0.96). Factor weights in this model were high (*λ* > 0.70) with a low level of associated error in all items (*e* < 0.49). Due to the low relationship between the two factors (− 0.15), a fourth model of two unrelated factors was evaluated (model 2b). Table 2 shows that although this model has acceptable fit indices (RMSEA = 0.078 [IC90% 0.062 – 0.094]_;_ SRMR = 0.090; CFI = 0.97; TLI = 0.96), model 1a continues to show better fit indices. Therefore, the model of two related factors of nine items (2a) will be used for the other analyses.

**Table 4:** Adjustment indices and factorial weights of the different models of the scale

Model	Fit indices							Factor 1 (*λ*)					Factor 2 (*λ*)				
	*χ*^2^	*df*	*p*	CFI	TLI	SRMR	RMSEA [90%CI]	1	4	5	6	9	2	3	7	8	10
Model 1a	138.6	34	.000	.96	.95	.074	.081 [.067–.095]	.79	.88	.85	.86	.49	.71	.83	.84	.77	.72
Model 1b	148.9	35	.000	.96	.94	.106	.083 [.070–.097]	.79	.88	.85	.86	.49	.71	.83	.85	.77	.71
Model 2a	92.7	26	.000	.97	.96	.059	.075 [.059–.092]	.78	.88	.85	.86	-	.71	.83	.84	.77	.72
Model 2b	101.2	27	.000	.97	.96	.090	.078 [.062–.094]	.79	.88	.85	.86	–	.71	.83	.85	.77	.71

*Note*: 1a Two factors related to ten items, 1b Two unrelated factors with ten items, 2a Two factors related to nine items, *2b *Two unrelated factors with nine items

### Reliability estimates

In the total study sample, the dimensions of the presence of meaning (α) and the search for meaning (*α* = 0.86; *ω* = 0.87) and search for meaning (*α* = 0.88; *ω* = 0.88) present adequate reliability indices. Similarly, it occurs in the male sample: the presence of meaning (*α* = 0.92; *ω* = 0.92) and search for meaning (*α* = 0.86; *ω* = 0.86), and in the female sample: the presence of meaning (*α* = 0.90; *ω* = 0.90) and search for meaning (*α* = 0.89; *ω* = 0.89).

### Factorial invariance by sex

Table 5 shows that the factorial structure of the scale has shown evidence of being strictly invariant for the groups of men and women in the sequence of proposed invariance models: metric invariance (ΔCFI = 0.001; ΔRMSEA = − 0.007), scalar (ΔCFI = 0.000; ΔRMSEA =  − 0.002) y strict (ΔCFI =  − 0.003; ΔRMSEA =  − 0.001).

**Table 5:** Invariance models according to sex

Unidimensional model	*χ*^2^	*df*	*p*	SRMR	TLI	CFI	RMSEA	Δ*χ*^2^	Δdf	*p*	ΔCFI	ΔRMSEA
Men	53.83	26	.001	.059	.94	.96	.072	–	–	–	–	–
Women	64.03	26	.000	.062	.96	.97	.076	–	–	–	–	–
Invariance model												
Configural	118.95	52	.000	.060	.95	.961	.074	–	–	–	–	–
Metric	118.94	59	.000	.064	.97	.974	.067	4.21	7	.755	.001	− 007
Scalar	129.21	66	.000	.065	.97	.974	.065	9.44	7	.222	.000	− 002
Strict	135.78	75	.000	.065	.97	.971	.064	12.10	9	.207	− .003	− 001

*Note*: *χ*2 Chi square, *df *Degrees of freedom; *SRMR* Standardized root mean square residual, *TLI *Tucker-lewis index, *CFI *Comparative fit index; *RMSEA *Root mean square error of approximation; Δ*χ*2 Differences in chi square; Δ*df *Differences in degrees of freedom; Δ*RMSEA *Change in root mean square error of approximation; *ΔCFI *Change in comparative fix index

### Validity base on the relationship to other constructs

**Fig. 1:**
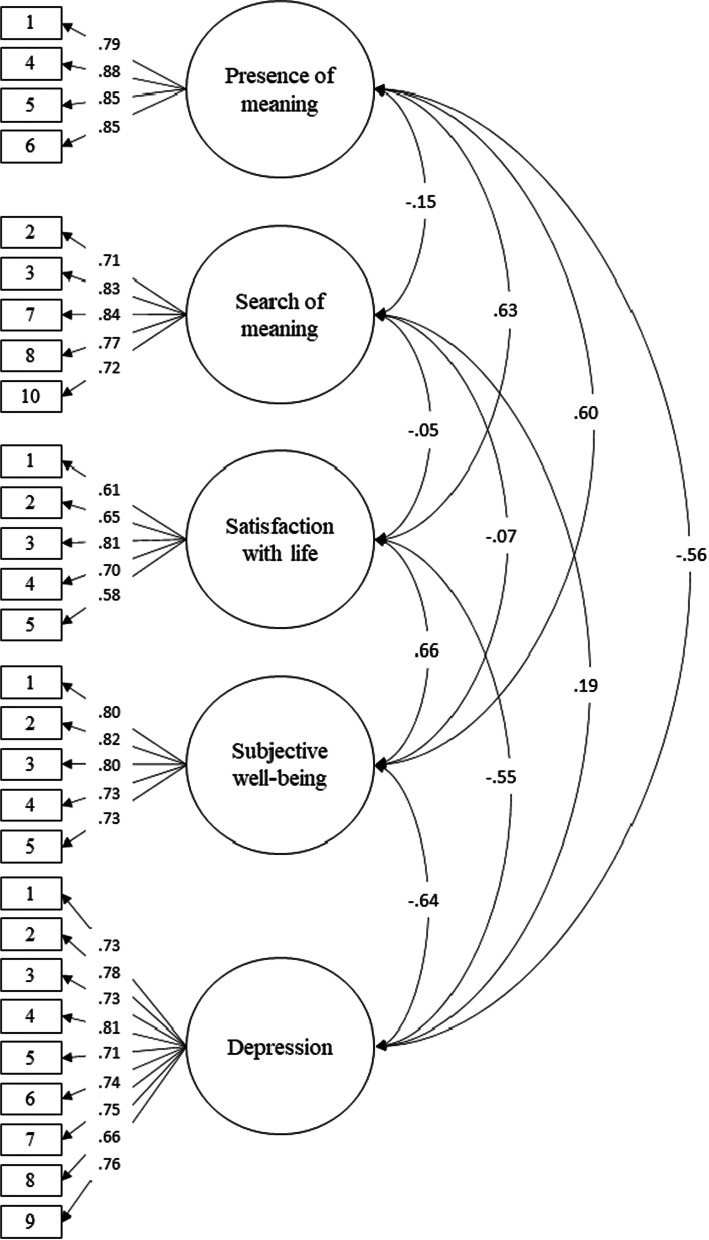
Relationship model with other variables

Considering the literature review, a Structural Equation Modelling (SEM) was proposed to evaluate the latent relationship between the MLQ scale and the level of satisfaction with life [19, 28, 35, 37, 55], well-being [33, 56, 58], and depression [19, 59]. It was evidenced that the model presents adequate fit indices (*χ*^2^ = 747.75; *df* = 340; *p* = 0.000; RMSEA = 0.049 [IC90% 0.044 – 0.054]; CFI = 0.95; TLI = 0.94). Figure 1 shows that the presence of meaning in life is significantly related to the level of satisfaction with life (0.63; *p* < 0.01), well-being (0.60; *p* < 0.01), and depression (− 0.56; *p* < 0.01). In contrast, the search for meaning in the life dimension is not significantly related to the level of satisfaction with life (− 0.050; *p* > 0.05) and psychological well-being (− 0.07; *p* > 0.05). However, it does maintain a significant relationship with the level of depression (0.19; *p* < 0.01). Considering these results, it can be concluded that the scale presents validity based on that related to other constructs.

## Discussion

The meaning of life is considered an essential factor that favors academic performance in university students [15]. About this, MLQ is one of the most used instruments for measuring this construct. However, in Latin America, no studies have yet been reported that have analyzed the psychometric properties of the MLQ in the university context. Therefore, the present study aimed to evaluate the psychometric properties of the MLQ in Peruvian university students.

Regarding the validity based on the internal structure, the results of the CFA show that model 2a presents adequate adjustment indices compared to the other models (1a, 1b y 2b). Models 1a and 1b values present adjustment problems in the indices of RMSEA (models 1a and 1b) and SRMR (1b). Also, item 9 ("My life does not have a clear purpose") is the only one that has a markedly negative connotation in the wording since it shows the lack of meaning in life. In this regard, it is important to specify that the presence of negative items decreases the reliability values [60–62], the variance of the scores decreases [62] and causes other additional factors in the factorial structure [62, 63]. This could explain why other psychometric studies of the MLQ conducted in the Spanish-speaking population found the presence of item 9 to be problematic [28, 64]. For all these reasons, it was considered appropriate to eliminate item 9. Subsequently, when evaluating model 2b, an excessive value was found in the SRMR, as well as a theoretical inconsistency, and the theoretical proposal of Steger et al. [6] indicates that both factors, the presence of meaning and the search for meaning, are related.

Given the above, model 2a was chosen since it presented adequate adjustment indices to the data. These findings of the two-dimensional structure are consistent with the theoretical proposal of Steger et al. [6], who points out that the meaning of life is explained by a cognitive factor that causes people to identify and understand its meaning from their experiences lived while the second motivational factor allows individuals to establish an active commitment to continue increasing their understanding about the meaning of life. Likewise, these findings coincide with previous studies carried out in Argentina [28], Ghana [37], Iran [36], United States [64] and Australia [33].

Regarding the relationship between presence and search for meaning, both dimensions show a low negative correlation, which is similar to that reported in studies carried out in countries such as Italy [35], Turkey [29], India [32], Argentina [28], United States [6], South Africa [19], and Australia [65]. However, it is different from the positive relationship reported in studies carried out in the countries of Nigeria [34], Iran [66], China [30], and Japan [27]. These differences in the relationship between both dimensions could be explained by cultural differences between countries since culture shapes the way people see themselves and their relationship with the world [67]. Also, culture influences how people develop and experience the presence and the search for meaning in life [68]. So cultural differences (individualism versus collectivism) and the resulting social and cognitive orientations (e.g., self-construction, open-mindedness) can influence the search for meaning in life. In this way, people from more collectivist cultures understand the search for meaning as a motivational force and a fundamental psychological need of the human being to understand his existence [1, 10], achieving a positive relationship between both factors. On the other hand, for people from more individualistic cultures, the search would mean an absence of meaning in life and an indicator of low personal development [8, 9, 56], which would explain a relationship negative between both factors.

Regarding reliability, the dimensions of the presence of meaning and search for meaning obtained good values in Cronbach's alpha coefficient. These findings are similar to those reported in other studies [28, 29, 34, 36, 57]. Similarly, it was found that both dimensions presented good values in the omega coefficient. It is important to note that this is the first study to show the internal consistency of the questionnaire through a more robust indicator, such as the omega coefficient [69]. Regarding the factorial invariance of the questionnaire, it was shown that the items could measure the meaning of life in men and women similarly and with the same precision. This result also suggests that the meaning of life measured through the MLQ is understood similarly by men and women. All this allows comparisons between both groups without measurement bias [70, 71]. This result is also consistent with the invariance findings in Brazil and China [31, 57].

Regarding the validity based on the relationship with other variables, it was found that the dimension of the presence of meaning in life obtained a positive relationship with life satisfaction and subjective well-being. These results are consistent with previous studies [28, 31, 35]. In addition, these results could be explained by the fact that the presence of meaning in life can lead an individual to develop moral self-acceptance while helping them find satisfaction in their lives, being a fundamental point to optimizing well-being [72]. Also, in the present study, a negative relationship between the presence of meaning and depression was evidenced. This result is consistent with previous literature since the presence of meaning in life is one of the most important factors in reducing the presence of depressive symptoms and promoting mental health indicators [13, 19].

On the other hand, it is shown that the search for meaning in life does not present a significant relationship with life satisfaction and subjective well-being. These results are different from those reported in studies conducted in South Africa [19], the United States [6, 58], Italy [35], Argentina [28] and Brazil [31]; where there is a negative relationship. This could be due to the moderating role of age; the literature mentions that for young people, the search for meaning can be normative and adaptive because they are exploring their identity. Similarly, studies show that with advancing age, there is a stronger association between the search for meaning and subjective well-being, since when the individual ages the search levels decrease [56, 73]. In this sense, having a sample of university students where most participants are young can explain the absence of association, unlike other studies with a greater age range. Likewise, the lack of relationship could be because this dimension can be experienced in different ways in people; that is, it can be understood as a factor of resilience, which motivates the individual to develop, or as an indicator of maladjustment or low personal development [58, 74]. On the other hand, the results show that the search for meaning is positively related to depression, consistent with previous studies [6, 19]. This relationship may be because people may consider the search a frustrating and challenging process that requires a deeper understanding of oneself and the world [75]. Finally, the observed relationships evidence the validity based on the relationship with other variables.

The study is not without limitations. In the first place, purposeful non-probabilistic sampling was carried out for the data collection, whose disadvantage is that it does not allow the generalization of the results. Therefore, it is suggested that future studies use probabilistic sampling. Second, the participants were 18 to 35 years, which limited the evaluation of the construct in other age groups. It is suggested that future studies expand the sample size and the age range for a greater understanding of the construct. Third, a longitudinal invariance was not performed to identify the temporal stability of the construct. Fourth, the test–retest reliability was not estimated, not allowing for evaluating the scores' temporal stability. Fifth, the construct was analyzed only by Peruvian university students. Therefore, cross-cultural studies are suggested to study whether the construct is similar in other Latin American countries.

Despite the limitations, this study showed that the MLQ has adequate psychometric properties from the perspective of CTT. In addition, the study provided evidence in favor of the two-dimensional model. Likewise, it was found that the scale is invariant in men and women, which will allow comparing the meaning of life between both groups without measurement bias. In addition, it was reported that the MLQ presents evidence of validity based on the relationship with other variables such as life satisfaction, well-being, and depression.

For all the above, the study provides the first psychometric evidence of the MLQ in the Peruvian university context, being the first study in Latin America, allowing the meaning of life to be adequately measured in university students.

## Data Availability

The datasets used and/or analyzed during the current study are available from the corresponding author on reasonable request.
